# A Systematic Review of the Prevalence and Diagnostic Workup of PIK3CA Mutations in HR+/HER2– Metastatic Breast Cancer

**DOI:** 10.1155/2020/3759179

**Published:** 2020-06-20

**Authors:** Elizabeth J. Anderson, Lea E. Mollon, Joni L. Dean, Terri L. Warholak, Ayal Aizer, Emma A. Platt, Derek H. Tang, Lisa E. Davis

**Affiliations:** ^1^University of Arizona College of Pharmacy, Tucson, AZ, USA; ^2^Harvard Medical School, MA, USA; ^3^Novartis, NJ, USA

## Abstract

PIK3CA mutation frequency varies among breast cancer (BC) subtypes. Recent evidence suggests combination therapy with the PI3K inhibitor (PI3Ki) alpelisib and endocrine therapy (ET) improves response rates and progression-free survival (PFS) in *PIK3CA*-mutant, hormone receptor positive (HR+) BC versus ET alone; thus, better understanding the clinical and epidemiologic elements of these mutations is warranted. This systematic review characterizes the *PIK3CA* mutation epidemiology, type of testing approaches (e.g., liquid or tissue tumor biopsy), and stability/concordance (e.g., consistency in results by liquid versus solid tumor sample, by the same method over time) in patients with HR+/HER2– advanced (locally unresectable) or metastatic disease (HR+/HER2– mBC) and explores performance (e.g., pairwise concordance, sensitivity, specificity, or predictive value) of respective mutation findings. A comprehensive search of PubMed/MEDLINE, EMBASE, Cochrane Central, and select conference abstracts (i.e., AACR, ASCO, SABCS, ECCO, and ESMO conferences between 2014 and 2017) identified 39 studies of patients with HR+, HER2– mBC. The median prevalence of *PIK3CA* mutation was 36% (range: 13.3% to 61.5%); identified testing approaches more commonly used tissue over liquid biopsies and primarily utilized next-generation sequencing (NGS), polymerase chain reaction (PCR), or Sanger sequencing. There was concordance and stability between tissues (range: 70.4% to 94%) based on limited data. Given the clinical benefit of the PI3Ki alpelisib in patients with PIK3CA mutant HR+/HER2– mBC, determination of tumor *PIK3CA* mutation status is of importance in managing patients with HR+/HER2– mBC. Prevalence of this mutation and utility of test methodologies likely warrants *PIK3CA* mutation testing in all patients with this breast cancer subtype via definitive assessment of PIK3CA mutational status.

## 1. Introduction

With an estimated 271,270 new cases in 2019, breast cancer (BC) is the most common nonskin cancer in women in the United States (US) [1]. Although most BC cases are diagnosed in the early stages, approximately 10 to 41% of patients develop metastatic or advanced (locally unresectable; stage 3 or 4) disease, depending on tumor characteristics and presentation [2]. The BC subtype known as hormone receptor positive, human epidermal growth factor receptor-2 negative (HR+/HER2-) represents 70% of cases [3]. The phosphoinositide 3-kinase (PI3K) pathway is the most frequently altered pathway in HR+ BC and is associated with tumor development, disease progression, and endocrine resistance [4]. The impact of *PIK3CA* mutation status on BC progression (e.g., localized to metastatic disease) is uncertain [5]. Current treatment options for postmenopausal HR+/HER2- advanced BC include endocrine therapy (ET) +/- a CDK 4/6 inhibitor, an mTOR inhibitor, or chemotherapy (CT) [6]. However, ET or TT+ET rather than chemotherapy constitutes the initial therapy usually administered for women with HR+ advanced BC; TT+ET has more manageable safety profiles than CT [7]. The National Comprehensive Cancer Network (NCCN) guidelines recommend that CT can be used [8] for patients where no clinical benefit is observed after 3 consecutive endocrine-based therapies (including ET and TT+ET) or for patients with symptomatic visceral disease. A growing body of research suggests that use of a phosphoinositide 3-kinase inhibitor (PI3Ki) in conjunction with ET may improve response rates and progression-free survival in *PIK3CA*-mutant, estrogen receptor positive (ER+) BC relative to ET alone [7, 9–12], precluding or delaying the need for CT. Additionally, the recent results of the SOLAR-1 Phase III trial provided evidence that the PI3Ki alpelisib given with fulvestrant, as opposed to placebo plus fulvestrant, improved PFS among patients with PIK3CA-mutated HR+/HER2- mBC who had received endocrine therapy previously [13], leading to FDA approval of alpelisib. The majority of patients in this trial had metastatic disease.

The frequency of *PIK3CA* mutations varies across different BC molecular subgroups [5]. One study found a 41.1% frequency of *PIK3CA* mutation in HR+/HER2- breast cancer compared to 12.5% of patients with triple negative breast cancer [14]. Previous evidence indicates that PI3Ki are active in postmenopausal women with *PIK3CA*-mutant HR+/HER2- advanced or metastatic breast cancer [12, 15, 16]; thus, detection of these *PIK3CA* mutations in tumors is important in identifying those patients most likely to benefit from treatment using a PI3Ki. Until recently, clinical guidelines did not recommend PIK*3CA* mutation testing as a part of standard testing (such as HR and HER2 status) [17, 18]. As such, the majority of testing has been performed by commercial next-generation sequencing (NGS) platforms and at institutions where in-house gene panels have been developed [19]. To date, the diagnostic yield (i.e., the proportion of patients in whom the testing technique yields a definitive diagnosis) of BC *PIK3CA* mutation testing has been challenging to measure, given the variability in prevalence of *PIK3CA* mutations throughout BC subtypes and lack of guidelines for testing in clinical practice [20]. Furthermore, a systematic understanding is lacking regarding the prevalence of *PIK3CA* mutations in HR+/HER2- advanced/unresectable or metastatic breast cancer or within clinically relevant molecular BC subgroups. Understanding the prevalence is important to support quantifying the size of the patient pool that may benefit from receiving *PIK3CA* testing.

Various biopsy and analytical testing approaches exist to detect *PIK3CA* mutations. However, evidence is lacking with respect to the real-world generalizability and applicability across tests due to the variations in test performance across approaches. Namely, tests for *PIK3CA* mutation have not been routinely performed in the clinical setting among patients with HR+/HER2- mBC, and concordance between testing methods (e.g., NGS vs. PCR), test location (e.g., primary site vs. metastasis), type of biopsy (e.g., liquid vs. tissue), or retest concordance (i.e., stability over time) are not well documented. Patients may undergo multiple tumor biopsies over time, particularly if the disease progresses during a specific treatment, and consequently, the test results will influence clinician decisions regarding subsequent therapy. Also, the tumor biopsy site may change over time. For example, tissue may be tested initially using archived tumor obtained at time of diagnosis, and due to a lack of a convenient site for new tissue biopsy, a liquid biopsy may be performed at a later time during therapy for metastatic disease [20, 21]. Demonstrating concordance (i.e., agreement in test results among testing methods) and stability (i.e., consistency in test results) between tests over time will reduce the clinical burden for both patients and providers by minimizing the need for repeat and/or invasive testing. Further, high concordance between tissue and liquid biopsy test results could lead to better convenience in patient care based on the availability of different testing technologies in various global clinical settings. Discordance between samples analyzed for gene mutations using distinct methods from the same or other sources of tumor DNA could be minimized if differences in sensitivities among testing methods are identified/understood.

The purpose of this systematic review was to describe the epidemiology of *PIK3CA* mutations and variation across respective *PIK3CA* testing methods among patients with *PIK3CA*-mutant or wild-type HR+/HER2- advanced or metastatic breast cancer. Additionally, this review is aimed at (1) describing the type of biopsy and analytical approaches for *PIK3CA* mutation testing and (2) assessing the performance (e.g., pairwise concordance, sensitivity, specificity, or predictive value between test types) and stability (e.g., consistency of result over time) of *PIK3CA* mutation findings.

## 2. Methods

### 2.1. Study Design

This review followed the Preferred Reporting Items for Systematic Review and Meta-Analyses (PRISMA) guidelines [22].

### 2.2. Participants, Interventions, and Comparators

At the level of titles and abstracts, we screened the following inclusion criteria: conducted with human subjects or human tissue, included a population or subpopulation with HR+/HER2- mBC, and reported information on the presence of the *PIK3CA* mutation. Status of cases as advanced or metastatic was accepted based on study authors' determination, but definitions for advanced cases were not universally available. Unless otherwise defined, advanced refers to those cases that are locally unresectable. Following title and abstract-level screenings, full-text articles and posters were reviewed for the following inclusion criteria: included patients with HR+/HER2- mBC, included either tissue or liquid biopsies, and reported *PIK3CA* mutation status among the HR+/HER2- mBC subgroup.

### 2.3. Systematic Review Protocol

The search strategy followed an *a priori* review protocol developed internally.

### 2.4. Search Strategy

A comprehensive search of PubMed/MEDLINE, EMBASE, Cochrane Central, and select conference abstracts (i.e., AACR, ASCO, SABCS, ECCO, and ESMO conferences between 2014 and 2019) was performed including, but not limited to, keywords: “breast neoplasm,” “*PIK3CA* protein,” “hormone receptor positive,” and “metastases” including all the search terms: text words (free text), subject index headings (e.g., MeSH), and the relationship between the search terms (e.g., Boolean). Full search strategy is available in Appendix A. Databases were searched on August 28, 2017, for English-language publications of patients with HR+, HER2– mBC published between January 1993 and April 2019 (with the last search occurring April 2019). The initial search performed in 2017 was updated in 2019 using both the comprehensive search terms described in Appendix A plus hand searching of new conference abstracts as well as the acronyms for clinical trials ongoing as of 2017 that were identified in the initial search (e.g., BELLE-3).

### 2.5. Data Sources, Study Sections, and Data Extraction

Data extraction methods followed Cochrane guidelines for systematic literature reviews [23]. Two independent researchers screened titles and abstracts resulting from all searches to identify potentially eligible studies. A full-text review was then performed by two independent researchers; any discrepancies were settled by an independent third researcher. Data extraction from full publication texts was performed by two independent researchers. The following data were compiled in a standardized table and validated by one or more other authors: study design, study country, number of participants and samples, participant demographics, testing methods, analytical approach and corresponding manufacturer data, mutations detected using corresponding analytical approach, primary tumor or metastasis location and size, frequency of mutation, concordance between testing sites or testing method as reported, and stability over time (Table 1). Where multiple reports were published based on the same study, frequency data were not duplicated but, rather, extracted from the most comprehensive publication. No contact with study authors was necessary.

Among full-text reports or posters considered for inclusion, the following criteria were used to determine if *PIK3CA* prevalence was reported: proportion of HR+/HER2- samples with *PIK3CA* mutation, number of samples tested for *PIK3CA* hotspot mutation, or performed a subgroup analysis by mutation status. To perform subanalyses relating to the prevalence of the *PIK3CA* mutation, the testing types utilized, and concordance between tests, studies meeting the initial inclusion criteria were subjected to additional specific inclusion criteria (e.g., relative to subanalysis) and categorized accordingly.

Concordance data related to the following factors were extracted: between analytical approaches (i.e., PCR vs. NGS vs. Sanger sequencing), between tissue samples (presence of mutations in primary tumors versus those from metastatic lesions), and between biopsy methods (i.e., tumor tissue biopsy versus liquid biopsy) when the assessment was done at a variety of time points, including time of initial diagnosis and time at disease progression. When available, we sought to compare results obtained using different modalities of testing obtained at the same time during a patient's oncologic course, the same modality of testing obtained over different times during a patient's oncologic course, and different modalities of testing obtained at different times over a patient's oncologic course.

### 2.6. Data Analysis

Included studies were assessed using content analysis, a systematic technique for describing data and outcomes using qualitative methods [24] and reports of prevalence. Descriptive statistics on the overall prevalence as well as prevalence for these subgroups considered clinically important by the clinical experts on this study team were computed: geographic location (US vs. international), study design (clinical trial vs. observational research), testing approaches (including type and location of sample tested and type of analytical testing method employed), and the manufacturer of the test. Among these subgroups, independent group *t*-tests were performed to measure difference in the mean prevalence of the *PIK3CA* mutation. Considering the wide range of study types and study designs included in the review, no meta-analysis was conducted.

The risk of bias for each study included for full-text review was independently completed by two authors using the Mixed Methods Appraisal Tool (MMAT, [25], Table 2). This tool was selected given the variety of study types, using both clinical and observational data, considered in this report. The risk of bias results were used to contextualize the relative strength of each included publication but did not influence inclusion or exclusion of studies. The MMAT has been previously evaluated for content validity and methodological quality [26, 27].

## 3. Results

**Figure 1:**
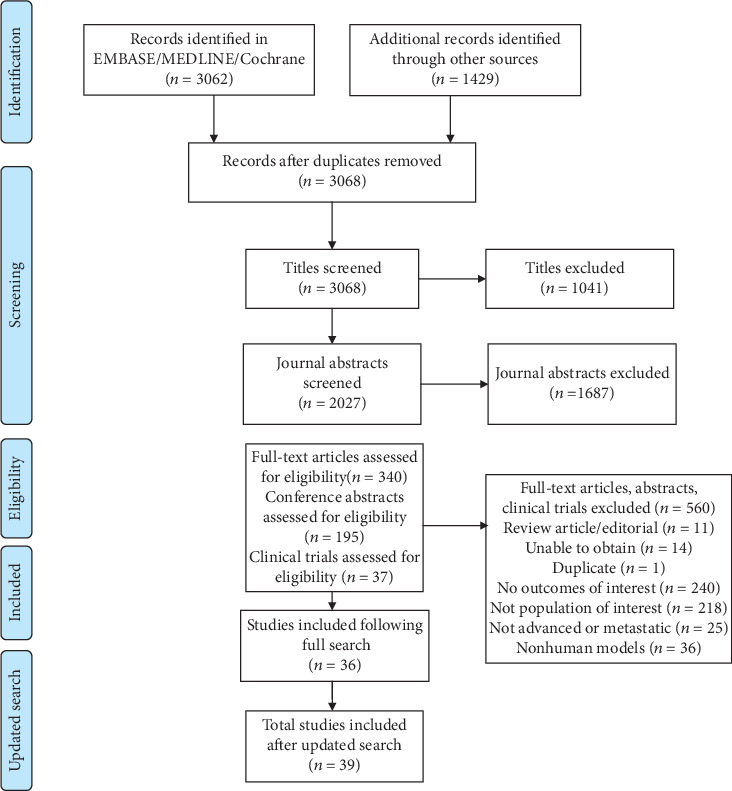
PRISMA flow diagram of article eligibility for inclusion.

Of 3068 articles and conference abstracts identified, 572 met the inclusion criteria and were included in a full-text review, of which 39 were included. Full study selection process is shown in Figure 1.

### 3.1. Study Selection and Characteristics

Prevalence of the *PIK3CA* mutation among HR+/HER2- mBC was reported in 37 of 39 studies that met the inclusion criteria (Table 1). Two studies (both presenting results of the SOLAR-1 Phase 3 trial) were enriched for patients with *PIK3CA*-mutated tumors and were excluded from prevalence estimation. Eighteen studies were observational in nature, and fifteen were performed in the US; twelve studies were conducted internationally, and twelve included both US and international populations. Across studies, a total of 6825 nonduplicated individual samples were tested for genetic mutations including *PIK3CA* (inclusive of samples from SOLAR-1, *n* = 572). The majority of included studies performed genetic testing using tissue biopsy samples (*n* = 36/39; 92.3%); tissue samples accounted for 73.1% of all samples across all studies (*n* = 4992). Liquid biopsies were performed in 9 studies, accounting for 26.7% of all samples (*n* = 1829). Six studies (four trials) reported both tissue and liquid biopsy data (Andre et al., 2019; Baselga et al., 2017; Board et al., 2010; Campone et al., 2018; Dickler et al., 2018; and Rugo et al., 2019). Among 15 studies that reported tumor histology, most samples were from ductal carcinomas (range: 51.5% to 96.1%). Across nine studies that reported tumor histology by *PIK3CA* mutation status, most were also from ductal carcinomas (range: 63.2% to 99%). The majority (*n* = 25) of studies included molecular data while 14 studies included data from 11 clinical trials. The sample sizes tested ranged from 9 to 618. Most studies did not specify which samples came from advanced versus metastatic cases so it was not possible to describe identified studies on these strata. At the level of study inclusion, cases were considered advanced based on study authors' determination though definitions for this term were not generally provided. Our baseline assumption for advanced BC was cases that were locally unresectable.

### 3.2. Prevalence

The overall reported prevalence of the *PIK3CA* mutation among all HR+/HER2-mBC samples ranged from 13.3% to 61.5% (median = 36.4%, IQR = 31.2%‐45.6%). More specifically, *PIK3CA* mutation prevalence ranged from 20.8% to 61.5% among tissue biopsies in this subgroup (*n* = 33 studies) and 43.4% to 46.8% among liquid biopsies (*n* = 6 studies). No statistically significant differences were observed in reported *PIK3CA* mutation prevalence by study location (i.e., U.S.-based vs. international) (mean difference = 0.035, *p* = 0.225), study type (i.e., clinical trial vs. observational) (mean difference = 0.0095, *p* = 0.836), or biopsy type (i.e., liquid or tissue) (mean difference = −0.033, *p* = 0.567); none of these variables varied by more than 3% between subgroups. Data from the SOLAR-1 phase 3 trial were excluded from the described prevalence estimates because it selectively enrolled a *PIK3CA*-mutant cohort and a *PIK3CA* wild-type cohort that did not reflect the likelihood of the mutation in the overall HR+/HER2- mBC population.

### 3.3. Hotspot Mutations

Hotspot mutations were reported in six of 39 studies; the most commonly reported among the HR+/HER2- mBC subgroup were H1047R and E545K. Frequency of the H1047R hotspot mutation ranged from 22% to 75% among identified *PIK3CA* mutations while the E545K mutation ranged from 11.1% to 50%. Other hotspot mutations identified were Q546K, H1047L, E542K, C420R, and N345. Of note, data from SOLAR-1 reported the following frequencies of hotspot mutations in their oversampled *PIK3CA*-mutant population: H1047X 57%; E542K 18%; E545K 31%. Although several additional studies reported the presence of hotspot mutations, they were not reported for the HR+/HER2- mBC subgroup specifically.

### 3.4. Testing Methodology and Analysis

The most common source of tumor DNA for identifying *PIK3CA* mutations among the HR+/HER2-mBC subgroup was tumor tissue biopsies (*n* = 36). Formalin-fixed paraffin-embedded (FFPE) tissue samples were used in most studies (*n* = 30) relative to other tissue or liquid sample types. Among liquid biopsies, two studies used circulating tumor cells (CTC), two studies used cell-free DNA (cfDNA), and seven studies (five trials) reported using circulating tumor DNA (ctDNA). Most studies reported using PCR for analysis (*n* = 28). Additional techniques included NGS (*n* = 11), Sanger sequencing (*n* = 5), and liquid chip technology (*n* = 2). Among clinical trials specifically (*n* = 12), PCR (*n* = 9), NGS (*n* = 1), and NGS plus Sanger sequencing (*n* = 2) were used. Four studies used multiple methods for analysis (Basho et al., 2016; Blackwell et al., 2015; Board et al., 2010; and Di Leo et al., 2017). Among studies using the NGS technique (*n* = 10), seven were cross-sectional and 3 were clinical trials. Additionally, ten of the eleven (90.1%) studies using NGS were published between 2015 and 2017.

### 3.5. Concordance

**Figure 2:**
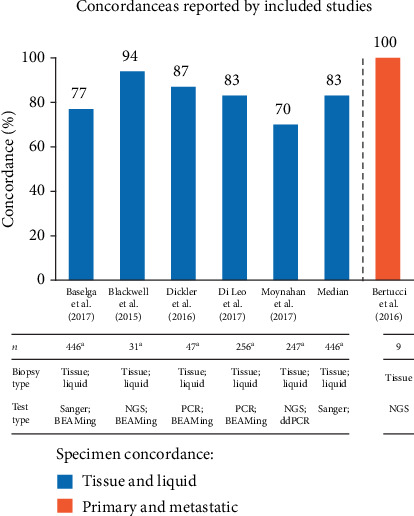
Concordance of *PIK3CA* mutation status as reported by included studies of HR+/HER2-mBC in clinical or observational trial patients.

Among the 39 studies that met the inclusion and exclusion criteria, only six reported data on concordance specific to the HR+/HER2-mBC subgroup (Figure 2). Of these six studies, concordance of PIK3CA mutations between tissue and liquid biopsies ranged from 70.4% to 94%. Studies generally did not report timing of sample collection (e.g., fresh or archival tissues), although some [10, 28, 29] specified whether samples from archival tissue were from primary or metastatic tumor, and one [9] reported that 3% of samples were from fresh biopsy. One study reported that subjects were enrolled according to local *PIK3CA* mutation testing; however, central testing for *PIK3CA* mutation was performed but did not report the concordance between the test results [30]. One study (*n* = 9 participants) reported 100% concordance between the mutation status of primary breast tumors and metastatic lesions [29]. No analysis of stability over time was performed due to insufficient information. For example, in the BOLERO-2, BELLE-2, and BELLE-3 trials, cfDNA or ctDNA was collected at randomization for *PIK3CA* mutation analysis and compared to results from archival tissue; however, only one trial reported concordance by tissue source [9, 10, 28]. In BELLE-2, 21% of patients who had PIK3CA-wild-type tumor tissue at randomization had evolved to PIK3CA-mutant status via ctDNA collected at a later time point, though the reference period is not reported [31]. In BOLERO-2, concordance of ctDNA against tissue biopsy was numerically higher among metastatic (*n* = 49) lesions (81.6%) relative to using all tissue samples (either primary or metastatic lesions) (70.4%) [28]. Variations in modalities of testing obtained at one or more time points in a patient's oncologic course were not reported in any of the six studies describing concordance.

### 3.6. Risk of Bias

The risk of bias for each included publication was stratified by study type per the MMAT (Table 2). Ten publications were rated as moderate to high risk of bias (i.e., did not meet all criteria), with 26 publications rated as low risk of bias (i.e., met all criteria). The risk of bias was performed at the publication rather than at the study level; hence, for those publications (e.g., posters and abstracts) where reported information was limited and a reduced MMAT score was assigned, poor study design (bias) is not necessarily implied. Including gray literature introduces additional bias given that such publications are not peer reviewed. Further, this tool does not account for potential publication bias nor selective reporting across studies that could influence the cumulative evidence given the inclusion of clinical trials.

## 4. Discussion

Increasingly, systemic therapy in patients with metastatic cancer is being driven by actionable mutations and rearrangements present within tumor tissue. In patients with metastatic non-small-cell lung cancer, for example, testing for EGFR mutations and ALK rearrangements, among other alterations, is critical for selecting the appropriate systemic therapy [32]. Among patients with mBC, systemic treatment has historically been guided by HR and HER2 status; the role of tumor profiling beyond HR/HER2 was less clear. More recently, however, the importance of testing for BRCA and PIK3CA in selected patients with breast cancer has been established by the utility of PARP inhibitors and the PI3Ki alpelisib in selected subpopulations, respectively. In the case of alpelisib, the SOLAR-1 study established improved PFS with alpelisib given with fulvestrant, as opposed to placebo plus fulvestrant, in patients with metastatic, PIK3CA-mutated, HR-positive, HER2-negative advanced or metastatic breast cancer [33], leading to FDA approval of the drug. The results of SOLAR-1 highlight the tangible benefit of testing for PIK3CA mutations in patients with HR+/HER2- mBC, an approach recommended by the NCCN consensus guidelines as well [8]. Various clinical and molecular studies have considered the epidemiological prevalence and clinical significance of *PIK3CA* mutation in BC using a variety of testing methods. The presence of a *PIK3CA* mutation is common in BC; however, the associated prognostic and predictive value is uncertain in the current clinical context due to variations in the source of tumor DNA, times when samples are obtained for testing during the disease course, and analytical methodologies and their respective sensitivity/specificity, as well as which hotspots are detected by the test. Our systematic review reports on 5701 advanced/locally unresectable or metastatic BC samples tested for *PIK3CA* mutation, primarily derived from retrospective analyses of tissue from archived samples, acquired as a part of observational or clinical trials. There is an increasing trend towards conducting clinical trials that test for the presence of genetic mutations up front (at initial diagnosis), yet this typically requires fresh tissue biopsies. However, published data regarding *PIK3CA*mutations in HR+/HER2– tumors obtained at diagnosis of metastatic disease is limited. Most samples were analyzed from tissue archived from primary tumor and a minority from metastatic tissue biopsies. Tumor DNA from liquid biopsies in some studies was collected with a paired tissue sample, though collection may have occurred at a different time. Since tumor *PIK3CA* mutation status has been shown to change over time in response to various lines of treatment, better understanding of the prevalence of *PIK3CA* mutation timeline is warranted, particularly in HR+/HER2– tumors [34, 35].

The median prevalence (36.4%) of *PIK3CA* mutation among patients with HR+/HER2– mBC, based on included literature, was similar to an estimated rate of 30% among all BC patients [5]. This estimated prevalence among the population of interest varied across testing techniques (i.e., tissue vs. liquid biopsy methods), although there was heterogeneity of reporting methods across studies. Differences in *PIK3CA* mutation prevalence by study site, biopsy type, and study type were not statistically significant. However, *PIK3CA* mutations and mutation hotspots (specifically H1047R and E545K) frequently occur in HR+/HER2– mBC. Given the potential prognostic and predictive value of *PIK3CA* mutation for both courses of treatment and overall survival, future research on the mutation is warranted for both this and other BC subtypes. Also, given differences in outcomes among intrinsic molecular subtypes of HR+ tumors (e.g., luminal A and luminal B), the implications of *PIK3CA* mutation with regard to these populations warrant consideration [36].

Most hotspot mutations among those with HR+/HER2– mBC were reported in H1047R and E545K and exist in exons 20 and 9, respectively. This finding is consistent with prior literature suggesting that *PIK3CA* mutations in these two exons are functionally important as oncogenes in breast cancer [37]. Specifically, exon 9 mutations are situated in the helical domain of p110*α* enabling it to avoid inhibitory regulation by p85, and thus, PI3K becomes an active kinase catalyzing conversion of PIP2 to PIP3. Consequently, subsequent cell signaling leads to increased growth, antiapoptosis, cell-cycle progression, and translation [5, 38]. Exon 20 mutations are situated in the kinase domain; however, the mechanism by which they confer PI3K signaling is not well understood [37]. A study by Lai et al. demonstrated that *PIK3CA* mutations were present in 26% of patients with invasive ductal breast carcinomas, with more than half of those occurring in exon 20. They further concluded that mutations in exon 20 were an independent risk factor of poor prognosis although any association with BC stage is not reported [39]. Our findings suggest that *PIK3CA* mutations in these hotspots remain present among the HR+/HER2- mBC subtype, which could have important prognostic value. However, literature reporting *PIK3CA* hotspot mutations among this important subgroup is lacking, and the sample sizes of those included studies reporting hotspot frequencies were mostly small.

This review reflects the most up-to-date summary of testing methods used to identify *PIK3CA* mutations associated with HR+/HER2- mBC. Despite the introduction and availability of newer, more precise analytical technologies such as NGS, the majority of studies still utilized PCR to test for *PIK3CA* mutation, using both tissue samples and liquid biopsies. However, NGS methodology was used more frequently in recent publications from both clinical and molecular trials, suggesting an overall trend towards the newer sequencing method. This discrepancy between PCR and NGS may be problematic for the prevalence of *PIK3CA* mutation comparisons in the population of interest given that the relative concordance between the two is not fully understood. Given the better suitability of NGS for high-throughput testing requirements with large clinical trials and commercial genomic profiling, *PIK3CA* mutation testing using PCR or Sanger sequencing may become less frequent [40]. However, recent clinical trials have used PCR to determine *PIK3CA* mutation status [10, 41]. While data were limited for measures of concordance across time and type of analysis, available evidence suggested a high level of concordance across sampling methods. Both clinicians and research investigators lack consistent approaches regarding if or how to retest for *PIK3CA* mutations at different time points or between different testable materials. This lack of protocol represents a significant gap in knowledge for the clinical applicability of *PIK3CA* testing for HR+/HER2– mBC patients. Additional evidence is needed to increase study generalizability as well as assess other possible confounding factors, such as differential timing between tumor tissue and liquid biopsies.

### 4.1. Limitations

This systematic review had several limitations. The included studies were limited to those published in English and those published by 2019. The included studies were further limited to those that specifically reported on cases of HR+/HER2- mBC and therefore do not necessarily represent all tests being implemented in *PIK3CA* research nor are they necessarily generalizable to other BC subtypes. Most studies only reported tests at a single time point or were cross-sectional samples of a larger study and did not necessarily describe when the tests were performed, if tests were performed more than once, or report on the reliability of the tests. In regard to analyses of concordance, the number of included studies was limited by search criteria used to estimate general prevalence of *PIK3CA* mutation, increasing subjection to reporting bias, and uncertainty around study findings. Heterogeneity in outcome reporting across studies limited consistency in the data presented.

## 5. Conclusions

This review emphasizes the significance of *PIK3CA* prevalence and mutation testing methodologies of HR+/HER2– mBC and the need for clinician familiarity with the relative value of available molecular tests in the context of targeted therapy. Routine *PIK3CA* testing from tissue or ctDNA isolated from plasma samples from patients with HR+/HER2– advanced/mBC is now recommended, as patients with PIK3CA-mutated breast cancer may be appropriate candidates for alpelisib, a recently FDA approved selective inhibitor of the *α* isoform of PI3Ki [42]. The use of alpelisib in combination with fulvestrant improved progression-free survival in patients with tumors harboring a *PIK3CA* mutation, which is associated with eventual endocrine resistance that occurs in advanced/metastatic disease [13]. Determinations of tumor *PIK3CA* mutation status may have additional implications for future research or treatment strategies [43].

## Figures and Tables

**Table 1:** Study characteristics, prevalence of *PIK3CA* mutations, and reported hotspots.

Authors	Year	Country	Number of participants with HR+/HER2- BC	Age^1^	Type of biopsy	HR+/HER2- *PIK3CA* wild type	HR+/HER2- *PIK3CA* mutants	Prevalence of PIK3CA mutants	HR+/HER2- *PIK3CA* hotspots^2^
			*n*	Median (range)		*n*	*n*	%	(*n*)
Abramson et al. [44]	2014	US only	110	52	Tissue	71	39	35.5%	
Andre et a1. [13]	2019	US including international	572	63 (25-92)	Tissue	231	341	NA^3^	E542K (60); E545X (105); H1047 (193); C420 (6); Q546 (5)^3^
Rugo et al. [33]^a^	2018		284		Liquid	115	169	NA^3^	
Ahmad et al. [45]	2016	International excluding US	89	50	Tissue	67	22	24.7%	H1047R (13); H1047L (1); E542K (2); E545A (1); E545G (1)
Arthur et al. [43]	2014	International excluding US	50	68 (25-94)	Tissue	24	26	52.0%	
Baselga et al. [9]	2017	US including international	1147	62	Liquid	387	200	34.1%	
					Tissue	584	276	32.1%	
Campone et al. [31]^b^	2018		1147						
Basho et al. [46]	2016	US only	343	47 (23-74)	Tissue	236	107	31.2%	
Baird et al. [47]	2016	International excluding US	30	53 (35-81)	Liquid	26	4	13.3%	H1047R (3); E545K (1)
Basu et al. [48]	2017	US only	11	n/a	Tissue	7	4	36.4%	E545K (2) H1047R (2)
Beelen et al. [49]	2014	International excluding US	41	n/a	Tissue	308	153	33.2%	Exon 20 (82), exon 9 (71)
Bertucci et al. [29]	2016	International excluding US	9	41 (33-72)	Tissue	4	5	55.6%	N345K (1); C420R (1); E542K (2)
Blackwell et al. [50]	2015	US including international	72	n/a	Tissue	37	18	32.7%	E545K (1); Q546K (1); H1047R (10); H1047L (2)
Board et al. [51]	2010	International excluding US	64	n/a	Liquid, tissue	33	10	28.00% (L) 23.26% (T)	H1047R (4); H1047L (2); E545K (6); E542K (1)
Christgen et al. [52]	2013	International excluding US	46	n/a	Tissue	22	24	52.2%	
Dickler [30]	2016	US including international	60	62 (31-82)	Liquid	25	22	46.8%	
					Tissue	29	31	51.7%	
Di Leo et al. [10]	2018	US including international	432	61	Tissue	204	109	34.0%	
Filipenko et al. [53]	2017	International excluding US	263	n/a	Tissue	10	32	76.2%	E542K (19); E545K (2); H1047R (8); H1047L (3)
Fleming et al. [54]	2012	US only	21	n/a	Tissue	18	5	21.7%	
Gasch et al. [55]	2016	International excluding US	23	n/a	Liquid	13	10	43.5%	
Gonzalez-Angulo et al. [56]	2011	US only	37	48 (30-83)	Tissue	19	15	44.1%	
Henderson et al. [57]	2016	US only	237	n/a	Tissue	129	108	45.6%	H1047A (37); Glu542Lys (27); GLu545Lys (26); other (18)
Lefebvre et al. [58]	2016	International excluding US	143	55 (26-82)	Tissue	90	53	37.1%	
Liu et al. [59]	2015	US including international	44	56	Tissue	30	14	32.5%	
Mayer et al. [60]	2017	US only	26	53	Tissue	10	16	61.5%	
Mayer et al. [61]	2014	US only	51	55 (34-77)	Tissue	35	16	31.4%	
Moynahan et al. [28]	2017	US including international	550	61 (54-68)	Liquid	312	238	43.3%	H1047R (138); E545K (61); E542K (39)
Muller et al. [62]	2016	US only	13	61	Tissue	8	5	38.5%	
Oliveira et al. [63]	2016	US including international	91	56	Tissue	65	26	28.6%	
Roy-Chowdhuri et al. [64]	2015	US only	132	n/a	Tissue	82	50	38.0%	
Sakr et al. [65]	2014	US only	31	n/a	Tissue	16	8	33.3%	
Soucier-Ernst et al. [66]	2015	US only	28	56 (31-78)	Tissue	14	14	50.0%	
Vetter [67]	2014	US including international	618	n/a	Tissue	441	177	28.6%	
Wang et al. [68]	2015	US only	22	57 (32-79)	Tissue	19	5	20.8%	
Welt et al. [69]	2013	US including international	73	63	Tissue	13	4	23.5%	
Williams et al. [70]	2014	US only	39	n/a	Tissue	23	8	26.0%	
Yuan et al. [71]	2016	US only	16	54.5 (34-78)	Tissue	7	9	56.3%	
Yuan et al. [35]	2015	International excluding US	376	49	Tissue	261	115	30.6%	
Zhang et al. [72]	2014	International excluding US	90	47 (25-71)	Tissue	60	30	33.3%	H1047R (13); H1047L (4); E545K (9); E542K (4)

^1^Median age and range reported where available; ^2^hotspots as reported; not all studies assessed by hotspot or reported hotspot by ER+/HER2- status; ^3^Participants with confirmed PIK3CA mutations were intentionally overselected for this trial. US: United States; HR+/HER2- BC: hormone receptor positive/human epidermal growth factor receptor-2 negative breast cancer; *PIK3CA*: phosphatidylinositol-4,5-bisphosphate 3-kinase catalytic subunit alpha; L: liquid; T: tissue. ^a^Two reports on participants in the SOLAR-1 phase 3 trial. ^b^Two reports on participants in the BELLE-2 phase 3 trial.

**Table 2:** Risk of bias assessment results using the Mixed Methods Appraisal Tool (MMAT).

Study	Study design	MMAT criteria met^1^				Risk of bias score^2^
		1	2	3	4	
Quantitative descriptive						
Abramson et al. (2014)	Observational	Y	Y	Y	Y	∗∗∗∗
Andre et al. (2018)^3^	Clinical	N	Y	Y	Y	∗∗∗
Ahmad et al. (2016)	Observational	Y	Y	Y	Y	∗∗∗∗
Arthur et al. (2014)	Observational	Y	Y	Y	Y	∗∗∗∗
Basho et al. (2016)	Clinical	Y	Y	Y	Y	∗∗∗∗
Basu et al. (2016)^3^	Observational	Y	Y	Y	Y	∗∗∗∗
Beelen et al. (2014)	Observational	Y	Y	Y	Y	∗∗∗∗
Bertucci et al. (2016)	Observational	Y	Y	Y	Y	∗∗∗∗
Board et al. (2010)	Clinical	Y	Y	Y	Y	∗∗∗∗
Campone et al. (2018)	Clinical	Y	N	Y	Y	∗∗∗
Christgen et al. (2013)	Observational	Y	Y	Y	N	∗∗∗
Dickler et al. (SABCS, 2016)^3^	Clinical	Y	Y	Y	Y	∗∗∗∗
Filipenko et al. (2017)	Observational	N	N	Y	Y	∗∗
Fleming et al. (2012)	Clinical	Y	Y	Y	Y	∗∗∗∗
Gasch et al. (2016)	Observational	Y	Y	Y	Y	∗∗∗∗
Gonzalez-Angulo et al. (2011)^3^	Observational	Y	Y	Y	Y	∗∗∗∗
Fitzgerald et al. (2016)	Observational	Y	Y	Y	N	∗∗∗
Lefebvre et al. (2016)	Observational	Y	N	Y	Y	∗∗∗
Liu et al. (2015)	Observational	N	Y	Y	Y	∗∗∗
Mayer et al. (2014)	Clinical	Y	Y	Y	Y	∗∗∗∗
Mayer et al. (2016)	Clinical	Y	Y	Y	Y	∗∗∗∗
Moynahan et al. (2017)	Clinical	Y	Y	Y	Y	∗∗∗∗
Muller et al. (2016)	Observational	Y	Y	Y	Y	∗∗∗∗
Oliveira et al. (2016)^3^	Observational	N	N	Y	N	∗
Roy-Chowdhuri et al. (2015)	Observational	Y	Y	Y	Y	∗∗∗∗
Rugo et al. (2019)^3^	Clinical	N	Y	Y	Y	∗∗∗
Sakr et al. (2014)	Observational	Y	Y	Y	Y	∗∗∗∗
Soucier-Ernst et al. (2015)^3^	Clinical	Y	Y	Y	Y	∗∗∗∗
Vetter et al. (2014)^3^	Observational	Y	N	Y	N	∗∗
Wang et al. (2015)^3^	Observational	N	N	Y	N	∗
Welt et al. (2013)	Observational	Y	Y	Y	Y	∗∗∗∗
Williams et al. (2014)^3^	Observational	Y	N	Y	N	∗∗
Yuan et al. (2015)	Clinical	Y	Y	Y	Y	∗∗∗∗
Yuan et al. (2016)	Clinical	Y	N	Y	N	∗∗
Zhang et al. (2014)	Clinical	Y	Y	Y	Y	∗∗∗∗
Quantitative randomized controlled						
Baselga et al. (2017)	Clinical	Y	Y	Y	Y	∗∗∗∗
Di Leo et al. (2018)	Clinical	Y	Y	Y	Y	∗∗∗∗
Quantitative nonrandomized						
Baird et al. (2016)^3^	Clinical	Y	Y	Y	Y	∗∗∗∗
Blackwell et al. (2015)	Clinical	Y	Y	Y	Y	∗∗∗∗
Dickler et al. (ASCO, 2016)	Clinical	Y	Y	Y	Y	∗∗∗∗

^1^Mixed Methods Appraisal Tool (MMAT) criteria available in Appendix B (corresponding with questions 1-4 for respective study type). ^2^Each ∗ indicates percentage (25%, 50%, 75%, or 100%) of criteria met where ∗ (25%) corresponds to a high risk of bias and ∗∗∗∗ (100%) corresponds to a low risk of bias. ^3^Study data reported in a poster or conference abstract. Y: yes; N: no.

**Table 3:** 

No.	Search String
1	exp breast neoplasms/ or exp breast cancer/
2	(breast$ adj3 (cancer$ or neoplas$ or oncolog$ or tumo?r$ or malignanc$ or carcinoma$ or adenocarcinoma$ or sarcoma$)).ti,ab.
3	(mammar$ adj3 (cancer$ or neoplas$ or oncolog$ or tumo?r$ or malignanc$ or carcinoma$ or adenocarcinoma$ or sarcoma$)).ti,ab.
4	(metasta$ or advance$ or second$ or recurren$ or inoperab$ or disseminat$ or incur$).ti,ab,sh.
5	(1 or 2 or 3) and 4
6	exp Breast/ and exp Neoplasm Metastasis/
7	(breast$ adj3 (metasta$ or advance$ or second$ or recurren$ or inoperab$ or disseminat$ or incur$)).ti,ab.
8	(mammar$ adj3 (metasta$ or advance$ or second$ or recurren$ or inoperab$ or disseminat$ or incur$)).ti,ab.
9	(breast$ or mammar$).ti,ab,sh.
10	((stage or grade or type) adj2 ("3" or III or "c" or "4" or "IV" or d)).ti,ab.
11	(N1 or N2$ or N3$ or pN1$ or pN2$ or pN3$).ti,ab,sh.
12	9 and (10 or 11)
13	OR/5-8,12
14	(Antineoplastic Agents). ti,ab,rn,kw.
15	(selective estrogen receptor modulators). ti,ab,rn,kw.
16	(estrogen antagonists). ti,ab,rn,kw.
17	(drug therapy). ti,ab,rn,kw.
18	(letrozole or Femara or CGS 20267 or CGS-20267 or 112809-51-5). ti,ab,rn,kw
19	(anastrozole or Arimidex or ZD1033 or ZD-1033 or ICI D1033 or 120511-73-1). ti,ab,rn,kw.
20	(exemestane or examestane or Aromasin or Aromasine or Aromasil or FCE 24304 or FCE-24304 or 107868-30-4). ti,ab,rn,kw.
21	(tamoxifen or Nolvadex or Novaldex or Soltamox or Tomaxithen or Zitazonium or ICI 46474 or ICI-46474 or ICI 47699 or ICI-47699 or 10540-29-1). ti,ab,rn,kw
22	(fulvestrant or Faslodex or ICI 182780 or ICI-182780 or ZM 182780 or ZM-182780 or 129453-61-8). ti,ab,rn,kw.
23	(palbociclib or Ibrance or PD 0332991 or PD-0332991 or 571190-30-2). ti,ab,rn,kw.
24	(everolimus or Afinitor or Certican or RAD001 or RAD 001 or SDZ RAD or SDZ-RAD or 159351-69-6).ti,ab,rn,kw.
25	(LEE011 or LEE-011 or Ribociclib or 1211441-98-3). ti,ab,rn,kw.
26	(abemaciclib or LY2835219 or LY2835210 or 1231929-97-7).ti,ab,rn,kw.
27	(capecitabine or Xeloda or 154361-50-9).ti,ab,rn,kw.
28	(doxorubicin or Adriamycin or Doxil or Adriablastin or Adriablastine or Adriblastin or Adriblastina or Adriblastine or Adrimedac or Doxolem or Doxorubicin or Doxotec or Farmiblastina or Myocet or Onkodox or Ribodoxo or Rubex 23214-92-8).ti,ab,rn,kw.
29	(paclitaxel or Abraxane or Paxene or NSC-125973 or NSC125973 or Anzatax or Onxol or Praxel or Taxol or 33069-62-4).ti,ab,rn,kw.
30	(docetaxel or Taxotere or Docefrez or RP 56976 or RP-56976 or 114977-28-5).ti,ab,rn,kw.
31	(cyclophosphamide or cytophosphane or Cytoxan or Endoxan or NSC 26271 or B 518 or B-518 or Cyclophosphane or Cytophosphan or Neosar or Procytox or NSC-26271 or 50-18-0).ti,ab,rn,kw.
32	(eribulin or Halaven or NSC 707389 or NSC-707389 or B 1793 or B 1939 or B-1793 or B-1939 or E 7389 or E-7389 or ER 086526 or ER-086526 or ER-86526 or ER086526 or eribulin or Halaven or 253128-41-5).ti,ab,rn,kw.
33	(ethinyl estradiol).ti,ab,rn,kw.
34	(megestrol acetate or Megace or Mestrel or Maygace or Megostat).ti,ab,rn,kw.
35	(methotrexate or Amethopterin or Trexall).ti,ab,rn,kw.
36	(Fluorouracil or 5FU or 5-FU or 5-Fluorouracil or 5 Fluorouracil or Fluoruracil or Adrucil or Carac or Efudix or Efudex or Fluoroplex or Flurodex or Fluracedyl).ti,ab,rn,kw.
37	(toremifene or Fareston or FC-1157a or FC 1157a or FC1157a).ti,ab,rn,kw.
38	(gemcitabine or Gemzar or LY 188011 or LY-188011).ti,ab,rn,kw.
39	(vinorelbine or Navelbine or KW 2307 or KW-2307).ti,ab,rn,kw.
40	(abraxane or Albumin Bound Paclitaxel or ABI007 or ABI-007 or ABI 007).ti,ab,rn,kw.
41	(ixabepilone or azaepothilone B or BMS247550 or BMS 247550 or BMS-247550 or Ixempra).ti,ab,rn,kw.
42	(cisplatin or cis-platinum or NSC-119875 or Platino or Platinol or Platidiam).ti,ab,rn,kw.
43	(carboplatin or Carbosin or Carbotec or Paraplatin or Carboplat or NSC-241240 or NSC 241240 or NSC241240).ti,ab,rn,kw.
44	(Fluoxymesterone or Stenox or Halotestin or fluoximesterone).ti,ab,rn,kw.
45	OR/14-44
46	(animals not humans).sh.
47	(comment or editorial or editorial or book or practice-guideline or letter or journal correspondence).pt.
48	45 not (46 or 47)
49	13 and 45 and 48
50	(Phosphatidylinositol 3-Kinases or “PIK3CA protein, human”)
51	49 and 50
52	limit 51 to english
53 4	remove duplicates from 52

**Table 4:** 

American Association for Cancer Research (AACR) (2014-2019)	“hormone receptor positive” OR “HR+” OR “ER+” OR “PR+” OR “estrogen receptor positive”
American Society of Clinical Oncology (ASCO) Breast Cancer Symposium (2014-2018)	breast cancer AND (“hormone receptor positive” OR “HR+” OR “ER+” OR “PR+” OR “estrogen receptor positive”)
San Antonio Breast Cancer Symposium (2014-2018)	breast cancer AND (“hormone receptor positive” OR “HR+” OR “ER+” OR “PR+” OR “estrogen receptor positive”)
European CanCer Organisation (ECCO) (2015-2016)	breast cancer AND (“hormone receptor positive” OR “HR+” OR “ER+” OR “PR+” OR “estrogen receptor positive”) Abstract title: breast cancer
European Society for Medical Oncology (ESMO) (2014, 2016)	breast cancer AND (“hormone receptor positive” OR “HR+” OR “ER+” OR “PR+” OR “estrogen receptor positive”) Abstract title: breast cancer

**Table 5:** 

Quantitative descriptive
Is the sampling strategy relevant to address the quantitative research question (quantitative aspect of the mixed methods question)?
Is the sample representative of the population understudy?
Are measurements appropriate (clear origin, or validity known, or standard instrument)?
Is there an acceptable response rate (60% or above)?

Quantitative randomized controlled (trials)
Is there a clear description of the randomization (or an appropriate sequence generation)?
Is there a clear description of the allocation concealment (or blinding when applicable)?
Are there complete outcome data (80% or above)? Is there low withdrawal/drop-out (below 20%)?
Is there low withdrawal/drop-out (below 20%)?

Quantitative nonrandomized
Are participants (organizations) recruited in a way that minimizes selection bias?
Are measurements appropriate (clear origin, or validity known, or standard instrument; and absence of contamination between groups when appropriate) regarding the exposure/intervention and outcomes?
In the groups being compared (exposed vs. nonexposed; with intervention vs. without; cases vs. controls), are the participants comparable, or do researchers take into account (control for) the difference between these groups?
Are there complete outcome data (80% or above), and, when applicable, an acceptable response rate (60% or above), or an acceptable follow-up rate for cohort studies (depending on the duration of follow-up)?

## Data Availability

The systematic review search strategy is available for replication.

## Appendix

### A. Search Strategy

#### A.1. MEDLINE, MEDLINE (R) In-Process, EMBASE CDSR, DARE, and CENTRAL (Ovid)

Tables 3 and 4 describe the search strategy used for databases and selected conference abstracts, respectively.

#### A.2. ClinicalTrials.gov

(letrozole OR Femara) AND (breast OR mammar∗)

(anastrozole OR Arimidex) AND (breast OR mammar∗)

(exemestane or Aromasin or Aromasil) AND (breast OR mammar∗)

(tamoxifen OR Nolvadex OR Soltamox) AND (breast OR mammar∗)

(fulvestrant OR Faslodex) AND (breast OR mammar∗)

(palbociclib OR Ibrance) AND (breast OR mammar∗)

(everolimus OR afinitor) AND (breast OR mammar∗)

(LEE011 OR LEE-011 OR Ribociclib) AND (breast OR mammar∗)

(abemaciclib) AND (breast OR mammar∗)

(capecitabine OR Xeloda) AND (breast OR mammar∗)

(doxorubicin OR Adriamycin OR Doxil) AND (breast OR mammar∗)

(paclitaxel OR Abraxane) AND (breast OR mammar∗)

(ethinyl estradiol) AND (breast OR mammar∗)

((Fluoxymesterone OR Stenox OR Halotestin OR fluoximesterone) AND (breast OR mammar∗)

(megestrol acetate OR Megace OR Mestrel OR Maygace OR Megostat) AND (breast OR mammar∗)

(docetaxel OR Taxotere OR Docefrez) AND (breast OR mammar∗)

(cyclophosphamide OR cytophosphane OR Cytoxan OR Endoxan) AND (breast OR mammar∗)

(eribulin OR Halaven) AND (breast OR mammar∗)

(methotrexate OR Trexall) AND (breast OR mammar∗)

(fluorouracil OR Adrucil OR Fluorouracil Novaplus OR PremierPro Rx Fluorouracil) AND (breast OR mammar∗)

(toremifene OR Fareston) AND (breast OR mammar∗)

(gemcitabine OR Gemcitabine Novaplus OR Gemzar OR PremierPro Rx Gemcitabine) AND (breast OR mammar∗)

(vinorelbine OR Navelbine OR Vinorelbine Novaplus) AND (breast OR mammar∗)

(paclitaxel OR paclitaxel protein bound OR Taxol OR Abraxane) AND (breast OR mammar∗)

(ixabepilone OR Ixempra) AND (breast OR mammar∗)

(cisplatin OR Platinol-AQ) AND (breast OR mammar∗)

(carboplatin OR Paraplatin OR Paraplatin NovaPlus OR) AND (breast OR mammar∗)

### B. Abridged Mixed Methods Appraisal Tool (2011) Criteria Used for Systematic Review

Table 5 notes the decision-making criteria for assignment of a quality score per the Mixed Methods Appraisal Tool.
